# Supplementary figures and images for: Circadian disruption and divergent microbiota acquisition under extended photoperiod regimens in chicken
Source: PeerJ. 2019 Mar 14;7:e6592. doi: 10.7717/peerj.6592 (PMC6421066; doi:10.7717/peerj.6592)

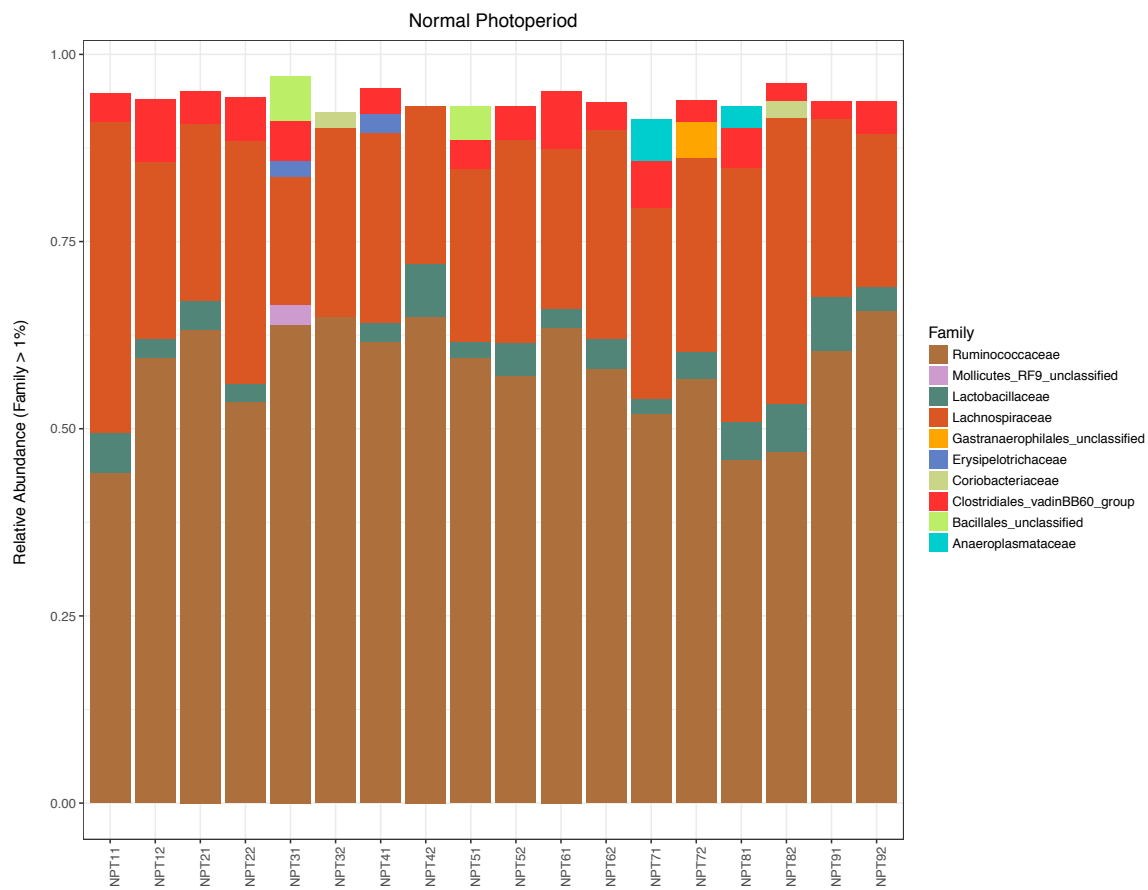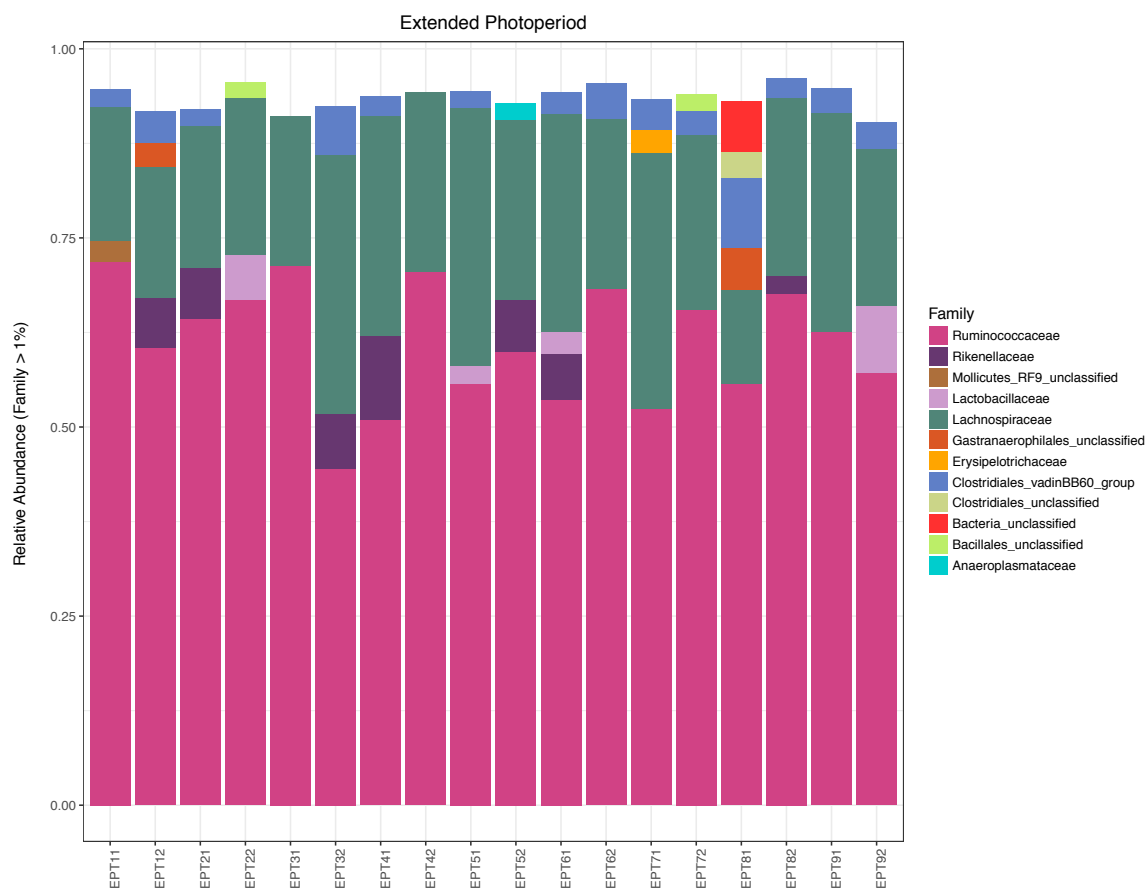

Supplement: Supplemental Information 1 — PCoA plot of microbial community from normal and extended photoperiod treatments. The plot shows the data against the second and third principal components, which explain about 22% of variation in the data. [file peerj-07-6592-s001.pdf]

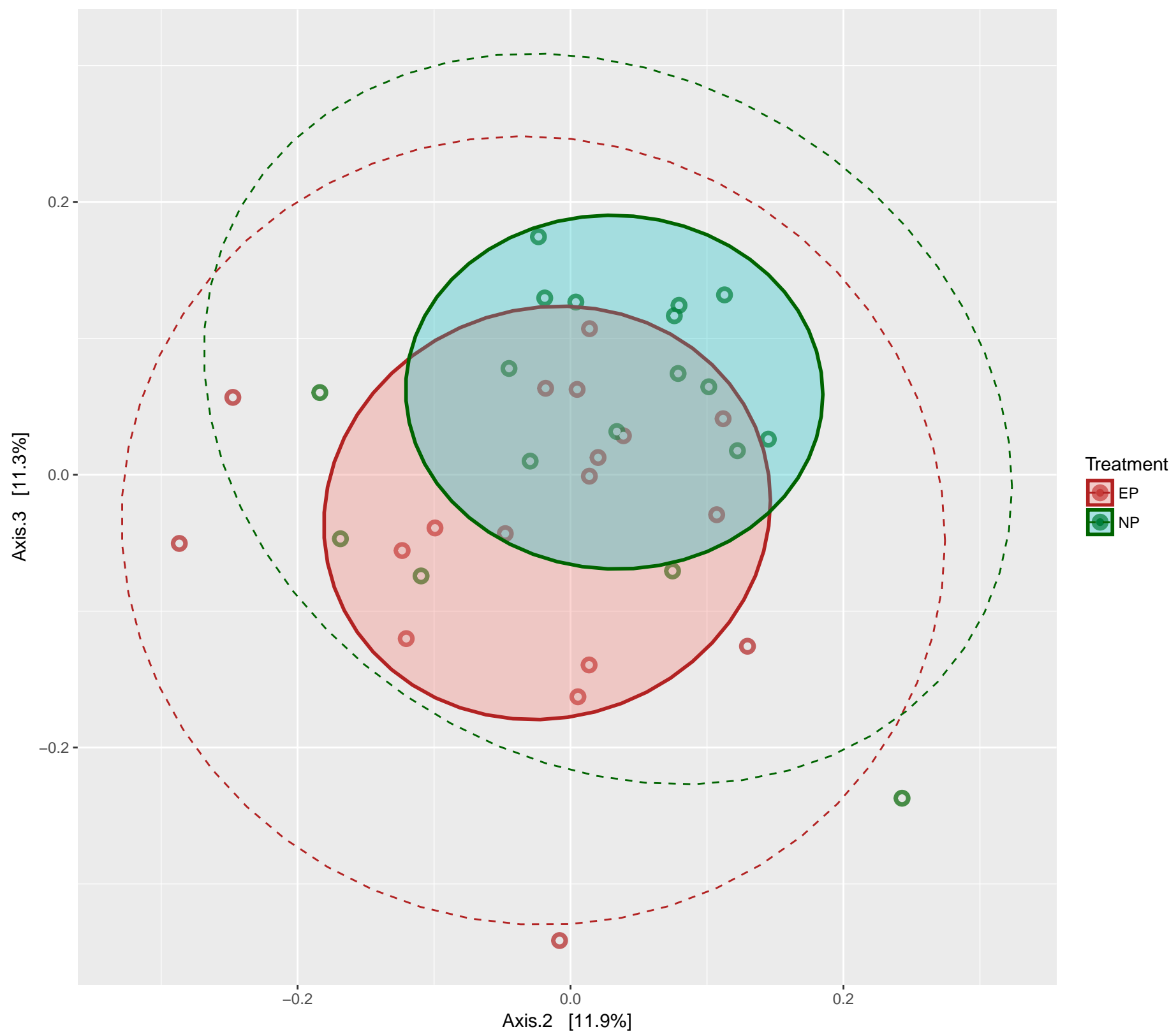

Supplement: Supplemental Information 2 — A network plot based on Bray-Curtis distance, showing the relative pairwise distances among individual samples belonging to either treatment. While this plot shows the differences in communities between two photoperiods, the communities still show a degree of overlap. [file peerj-07-6592-s002.pdf]

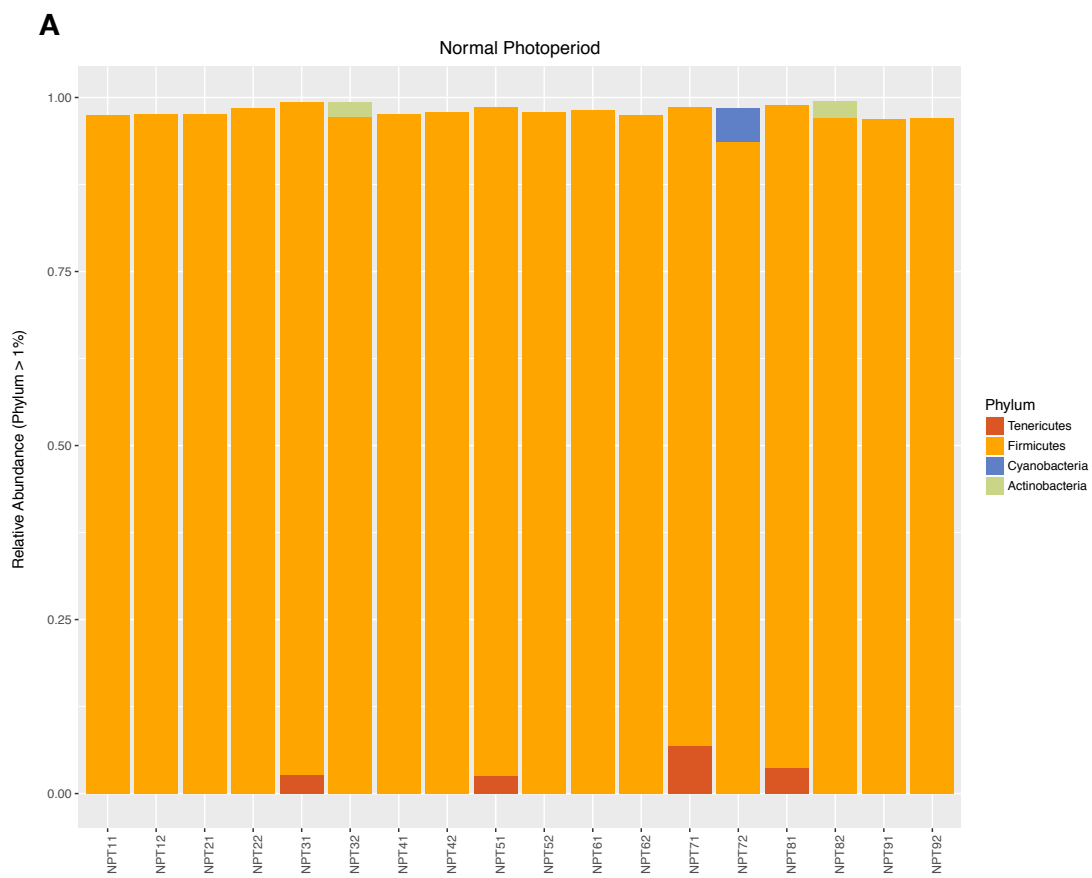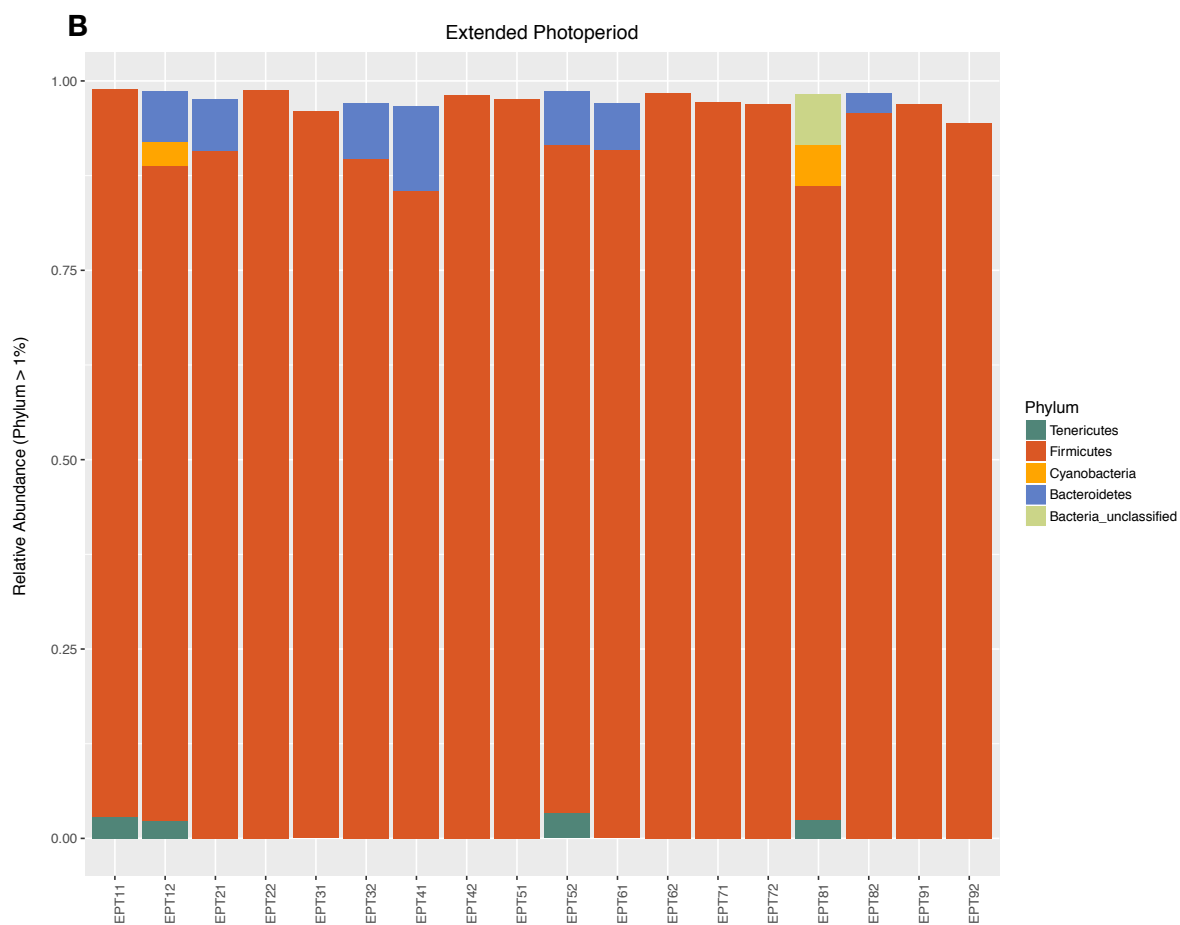

Supplement: Supplemental Information 3 — Figure showing column plots for phylum level classifications in the normal photoperiod (A), and extended photoperiods (B) treatments. Plots show Firmicutes to be the most abundant taxa in both treatments. [file peerj-07-6592-s003.pdf]

Phylum Composition

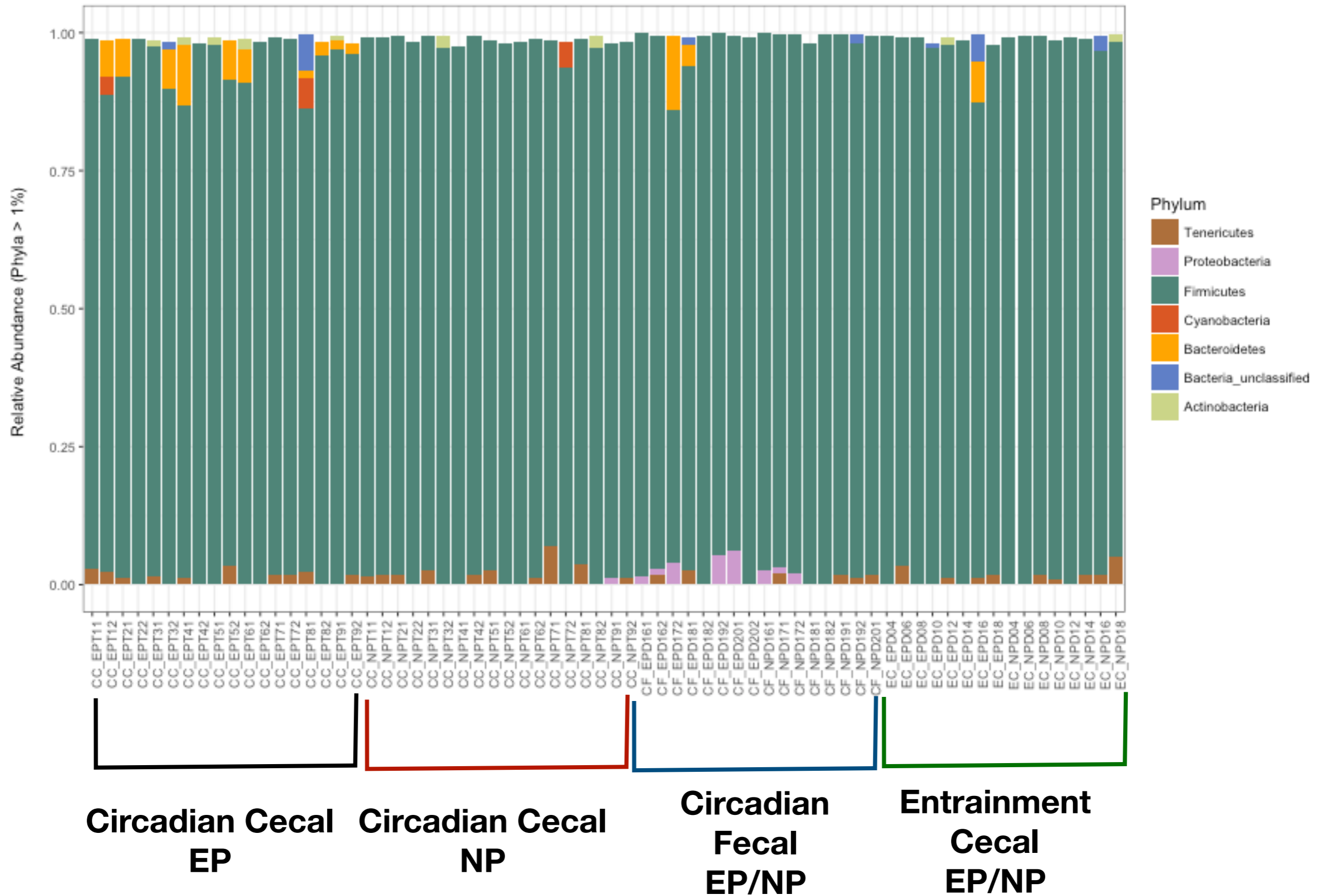

Supplement: Supplemental Information 4 — Column plot showing phylum level classification in all samples from across treatments. Different comparison groups are delineated using brackets on the bottom–namely, the EP cecal treatment, NP cecal treatment, Fecal microbiota samples (EP & NP), and the entrainment cecal group (EP & NP). [file peerj-07-6592-s004.pdf]
